# ADL dependence may represent a potential pathway linking chronic lung disease and depression in the middle-aged and older adults: A prospective cross-national cohort study (STROBE)

**DOI:** 10.1097/MD.0000000000049589

**Published:** 2026-07-03

**Authors:** Yu-Li Zhang, Lei Qin, Wen-Bin Bai, Nian Shen, Jing-Xia Qin

**Affiliations:** a Department of Interventional Radiology, The First College of Clinical Medical Science, China Three Gorges University, Yichang, Hubei Province, China; b Yichang Three Gorges Reservoir Area Ecological Environment Monitoring Station, Yichang Ecological Environment Monitoring Center, Yichang, Hubei Province, China.

**Keywords:** activities of daily living, China Health and Retirement Longitudinal Study, chronic lung disease, depression, English Longitudinal Study of Ageing, Health and Retirement Study, mediation analysis

## Abstract

Previous studies have indicated that middle-aged and older adults with activities of daily living (ADL) dependence or chronic lung disease (CLD) are more susceptible to depression. This study aimed to explore the potential mediating role of ADL dependence. Data from the 2015 to 2020 China Health and Retirement Longitudinal Study (CHARLS), the 2014 to 2019 English Longitudinal Study of Ageing (ELSA), and the 2014 to 2020 Health and Retirement Study (HRS) were utilized for analysis. Spearman correlation analysis was performed to detect correlations among CLD, ADL dependence, and depression. Logistic regression was used to adjust for covariates, and mediation analysis was performed using nonparametric bootstrap and multiple imputation. A total of 18,554 participants, 5,418 from CHARLS, 4560 from ELSA, and 8576 from HRS, were included in this study. The incident depression rates in the CHARLS, ELSA, and HRS cohorts were 27.5%, 11.3%, and 13.4%, respectively. Spearman correlation analysis demonstrated significant associations among CLD, depression, and dependence in both basic ADL and instrumental ADL (*P* < .05). Across the 3 cohorts, the potential mediating effect of basic ADL dependence on the CLD–depression link accounted for 7.8% in CHARLS, 13.7% in ELSA, and 10.1% in HRS. For instrumental ADL dependence, the potential mediating effect proportions were 6.2% in CHARLS, 9.1% in ELSA, and 12.1% in HRS, respectively. ADL dependence may partially mediate the CLD–depression relationship across diverse populations. However, the effect is weak, and the value of ADL interventions in preventing depression is limited.

## 1. Introduction

Depression is a serious mental disorder with a steadily increasing incidence worldwide. Over the past 30 years, its prevalence has risen by approximately 50%, affecting more than 264 million individuals globally.^[1]^ This upward trend is particularly pronounced among middle-aged and older adults. A previous study reported that the global prevalence of major depressive disorder in the elderly population is approximately 13.3%.^[2]^ In China, a national survey conducted in 2014 found that the prevalence of depression among individuals aged over 45 years exceeded 30%.^[3,4]^ Depression and chronic lung disease (CLD) frequently coexist, with a comorbidity prevalence ranging from approximately 10% to 65%.^[5]^ A meta-analysis demonstrated a negative correlation between depression and lung function.^[6]^ Compared with healthy individuals, patients with chronic obstructive pulmonary disease (COPD) had a higher risk of developing anxiety or depression.^[7]^ A meta-analysis of 79 studies from 25 countries worldwide demonstrated that patients with COPD have a 3.53-fold higher likelihood of depression compared with those without COPD, with approximately one-third of COPD patients experiencing depressive symptoms.^[8]^ CLD is often associated with activities of daily living (ADL) dependence. A study involving 1235 participants aged 60 and above from Southern and Central China demonstrated a positive correlation between CLD and the occurrence of ADL dependence (odds ratio [OR] = 2.034).^[9]^

ADL dependence is highly prevalent among older adults.^[10]^ ADL encompass both basic ADL (BADL) and instrumental ADL (IADL), which are commonly used indicators to assess functional independence in the elderly.^[11,12]^ BADL represent the ability to perform fundamental self-care tasks, whereas IADL represent the capacity to live independently within the community.^[13]^ BADL dependence has been associated with emotional changes, while IADL dependence tends to restrict social engagement, further exacerbating emotional disturbances and precipitating depression in this population.^[14]^ Several studies have demonstrated a significant association between ADL dependence and the occurrence of depression.^[15–18]^ The relationship between ADL dependence and depression is complex and bidirectional. However, ADL dependence caused by depression is relatively low, with depression more commonly arising from ADL dependence.^[18]^ A recent study indicated that improving ADL can reduce the occurrence of depression by 17% among the elderly population.^[14]^

Although existing research has elucidated associations between ADL dependence and depression,^[16]^ as well as between CLD and depression,^[19]^ the potential mediating role of ADL dependence remains poorly understood. Furthermore, there is a lack of comparative studies examining the mediating role of ADL dependence across different elderly populations. Therefore, in this study, we hypothesize that ADL dependence is a potential mediator between CLD and depression in middle-aged and older adults. We utilized 3 large national cohorts for analysis, including the China Health and Retirement Longitudinal Study (CHARLS), the English Longitudinal Study of Ageing (ELSA), and the Health and Retirement Study (HRS).

## 2. Methods

### 2.1. Study design and participants

**Figure 1.**
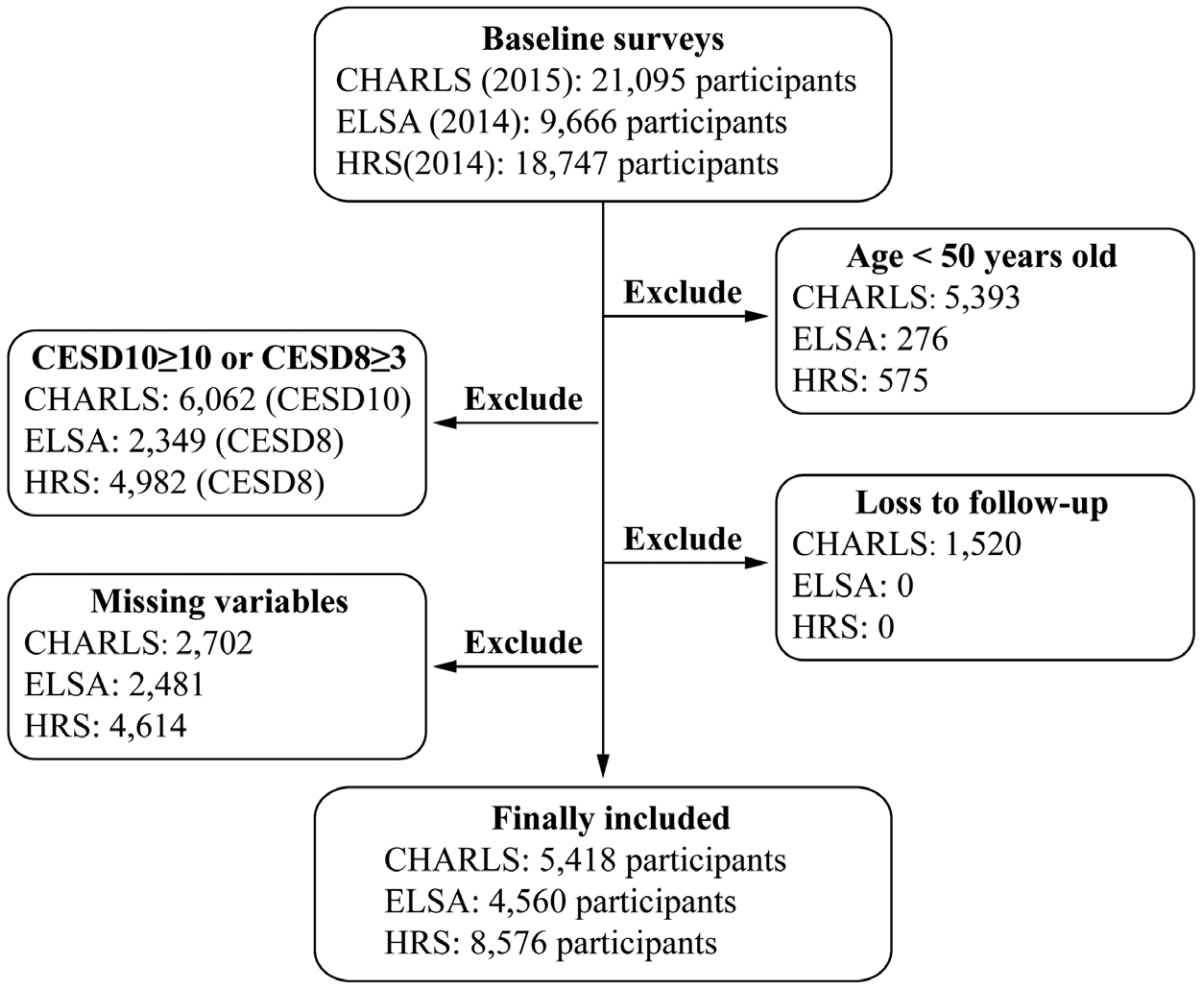
Flowchart of participant selection. CESD = Center for Epidemiologic Studies Depression Scale, CHARLS = China Health and Retirement Longitudinal Study, ELSA = English Longitudinal Study of Ageing, HRS = Health and Retirement Study.

The data for this study were obtained from CHARLS, ELSA, and HRS. CHARLS is a nationally representative survey database of middle-aged and older adults in China. CHARLS, ELSA, and HRS represent nationwide prospective cohorts from China, the UK, and the US, respectively. These cohorts comprehensively collect data on health status, lifestyle, and other relevant information among middle-aged and older adults. In this study, we utilized baseline data from CHARLS conducted in 2015, ELSA in 2014, and HRS in 2014. Following the exclusion of participants younger than 50 years, those defined as having depression, and individuals with missing variables, respondents were matched by their unique IDs to the corresponding follow-up data from CHARLS, ELSA, and HRS. The end of follow-up data was drawn from CHARLS in 2020, ELSA in 2019, and HRS in 2020. The participant selection process is illustrated in Figure 1.

### 2.2. Definition of CLD

In CHARLS, participants were asked the question, “Have you ever been diagnosed by a doctor with CLD, such as chronic bronchitis or emphysema (excluding tumors or cancer)?” Similarly, in ELSA, respondents were asked whether they had chronic bronchitis, emphysema, or COPD. Participants who answered “Yes” to these questions were classified as having CLD; otherwise, they were classified as non-CLD. In HRS, respondents were asked: “Has a doctor ever told you that you have CLD, such as chronic bronchitis or emphysema?” Similarly, CLD was defined based on the respondents’ answers.

### 2.3. Definition of depression

In the Chinese population, depression was assessed using the 10-item Center for Epidemiologic Studies Depression Scale, which has been widely applied in studies of depression among Chinese individuals.^[20,21]^ Following previous studies, individuals with a total score of 10 or higher were classified as having depression.^[17]^ In ELSA and HRS, depression was assessed using the 8-item Center for Epidemiologic Studies Depression Scale, consistent with prior research.^[22]^ Individuals with a total score of 3 or higher were defined as having depression.^[23,24]^

### 2.4. Definition of ADL and ADL dependence

BADL encompass the ability to perform tasks such as dressing, bathing, eating, transferring in and out of bed, toileting, and ambulating within a room.^[12]^ IADL include the capacity to carry out household chores, shopping, medication management, financial management, and cooking.^[12]^ Based on previous studies, participants were classified as having BADL dependence if they were unable to perform any 1 of the 6 specified items.^[14]^ Similarly, inability to complete any 1 of the 5 specified items was considered indicative of IADL dependence.^[14]^ In CHARLS, participants were not specifically asked about their ability to ambulate within a room. Therefore, self-control of defecation and urination was used as surrogate indicators for this activity.^[14]^

### 2.5. Covariates

Several covariates were selected, including age (≤60 years old and >60 years old), sex (male and female), marital status, educational level, history of smoking, history of alcohol consumption, hypertension, and diabetes.^[25]^ In CHARLS, ELSA, and HRS, marital status was categorized as married or other. Educational level was classified as high school or below versus college or above. History of smoking and drinking were dichotomized as yes or no. Hypertension or diabetes was defined as the use of antihypertensive or antidiabetic medications in combination with self-reported hypertension or diabetes diagnoses.

### 2.6. Statistical analyses

Continuous variables were presented as mean (standard deviation) or median (interquartile range) and compared between groups using the Wilcoxon rank-sum test or Student *t* test, as appropriate. Categorical variables were expressed as counts (n) and percentages (%), with group differences assessed using the chi-square test or Fisher exact test.

We first employed Spearman correlation analysis to assess the associations among the primary variables, including CLD, ADL dependence, and depression. Subsequently, logistic regression analyses were conducted as follows: examining the association between CLD and depression; analyzing the relationship between CLD and ADL dependence, including both BADL dependence and IADL dependence; and assessing the association between CLD and depression while incorporating BADL dependence and IADL dependence separately as mediators. All logistic regression models were adjusted for covariates. Nonparametric bootstrap resampling with 1000 iterations was applied to estimate total, direct, and indirect effects along with their 95% confidence intervals (CIs), and to calculate the proportion mediated.^[26]^ The mediation analyses were performed using the *mediation* package (version 4.5.1). To ensure the robustness of our findings, 20 imputations using the *mice* package (version 3.16.0) were performed to mitigate selection bias caused by missing data. Subsequently, mediation analysis was conducted on imputed data. All statistical analyses were carried out using R software (version 4.2.3; R Foundation for Statistical Computing). A two-sided *P* value <.05 was considered statistically significant.

### 2.7. Ethics approval

The CHARLS data were approved for public release after review by the Biomedical Ethics Committee of Peking University (IRB00001052-11015). The ELSA was approved by the London Multicenter Research Ethics Committee (MREC/01/2/91). The HRS was approved by the Institutional Review Board at the University of Michigan and the National Institute on Aging (HUM00061128). This study utilized publicly available data, all of which can be accessed on open websites. The research does not involve any nonpublic personal information of participants, and therefore, it does not require ethical review.

## 3. Results

### 3.1. Participant characteristics

A total of 18,554 participants were included in the study, comprising 5418 respondents from CHARLS, 4560 respondents from ELSA, and 8576 respondents from HRS. The demographic characteristics and health status across the different databases are presented in Table 1. Incident depression cases numbered 1491 (27.5%) in CHARLS, 515 (11.3%) in ELSA, and 1145 (13.4%) in HRS. In CHARLS, age differed significantly between the depression and non-depression groups (*P* < .001), whereas no significant age differences were observed in the ELSA and HRS cohorts (Supplementary Table S1, Supplemental Digital Content 1). Similarly, history of smoking showed statistically significant differences between groups in CHARLS and HRS (*P* < .05), but not in ELSA (Supplementary Table S1, Supplemental Digital Content 1). Analyses of other covariates yielded consistent results across the 3 databases. In addition, we compared the baseline characteristics of included and excluded participants, and details are provided in Supplementary Table S2, Supplemental Digital Content 2.

**Table 1:** The characteristics of participants in this study.

Variables	Total (n = 18,554)	CHARLS (n = 5418)	ELSA (n = 4560)	HRS (n = 8576)	*P* value
Depression, n (%)					<.001
Non-depression	15,403 (83)	3927 (72.5)	4045 (88.7)	7431 (86.6)	
Depression	3151 (17)	1491 (27.5)	515 (11.3)	1145 (13.4)	
Age, n (%)					<.001
≤60 yr	6646 (35.8)	2688 (49.6)	1117 (24.5)	2841 (33.1)	
>60 yr	11,908 (64.2)	2730 (50.4)	3443 (75.5)	5735 (66.9)	
Sex, n (%)					<.001
Female	9808 (52.9)	2457 (45.3)	2393 (52.5)	4958 (57.8)	
Male	8746 (47.1)	2961 (54.7)	2167 (47.5)	3618 (42.2)	
Marital status, n (%)					<.001
Married	9234 (49.8)	4887 (90.2)	1251 (27.4)	3096 (36.1)	
Other	9320 (50.2)	531 (9.8)	3309 (72.6)	5480 (63.9)	
Education status, n (%)					<.001
High school and below	11,259 (60.7)	5300 (97.8)	2209 (48.4)	3750 (43.7)	
College and above	7295 (39.3)	118 (2.2)	2351 (51.6)	4826 (56.3)	
Smoking status, n (%)					<.001
No	8618 (46.4)	2768 (51.1)	1815 (39.8)	4035 (47)	
Yes	9936 (53.6)	2650 (48.9)	2745 (60.2)	4541 (53)	
Drinking status, n (%)					<.001
No	7152 (38.5)	3333 (61.5)	474 (10.4)	3345 (39)	
Yes	11,402 (61.5)	2085 (38.5)	4086 (89.6)	5231 (61)	
Diabetes, n (%)					<.001
No	16,015 (86.3)	5130 (94.7)	4153 (91.1)	6732 (78.5)	
Yes	2539 (13.7)	288 (5.3)	407 (8.9)	1844 (21.5)	
Hypertension, n (%)					<.001
No	10,626 (57.3)	4107 (75.8)	2775 (60.9)	3744 (43.7)	
Yes	7928 (42.7)	1311 (24.2)	1785 (39.1)	4832 (56.3)	
CLD, n (%)					<.001
No	17,024 (91.8)	4792 (88.4)	4345 (95.3)	7887 (92)	
Yes	1530 (8.2)	626 (11.6)	215 (4.7)	689 (8)	
BADL, n (%)					<.001
Independence	16,786 (90.5)	4832 (89.2)	4115 (90.2)	7839 (91.4)	
Dependence	1768 (9.5)	586 (10.8)	445 (9.8)	737 (8.6)	
IADL, n (%)					<.001
Independence	17,128 (92.3)	4688 (86.5)	4382 (96.1)	8058 (94)	
Dependence	1426 (7.7)	730 (13.5)	178 (3.9)	518 (6)	

BADL = basic activities of daily living, CHARLS = China Health and Retirement Longitudinal Study, CLD = chronic lung disease, ELSA = English Longitudinal Study of Ageing, HRS = Health and Retirement Study, IADL = instrumental activities of daily living.

### 3.2. Correlation between CLD, ADL dependence, and depression

Spearman correlation analysis demonstrated that in all 3 datasets, CLD was positively correlated with BADL dependence (CHARLS: *r* = 0.062; ELSA: *r* = 0.080; and HRS: *r* = 0.090), IADL dependence (CHARLS: *r* = 0.038; ELSA: *r* = 0.073; and HRS: *r* = 0.078), and depression (CHARLS: *r* = 0.051; ELSA: *r* = 0.058; and HRS: *r* = 0.066, Table 2). Furthermore, BADL dependence (CHARLS: *r* = 0.093; ELSA: *r* = 0.118; and HRS: *r* = 0.100) and IADL dependence (CHARLS: *r* = 0.120; ELSA: *r* = 0.100; and HRS: *r* = 0.093) were also positively correlated with depression. All these associations were statistically significant (Table 2, *P* < .001).

**Table 2:** Correlations among chronic lung diseases, activities of daily living and depression.

	CHARLS				ELSA				HRS			
	CLD	BADL	IADL	Depression	CLD	BADL	IADL	Depression	CLD	BADL	IADL	Depression
CLD	1.000***	0.062***	0.038***	0.051***	1.000	0.080***	0.073***	0.058***	1.000	0.090***	0.078***	0.066***
BADL	–	1.000***	0.266***	0.093***	–	1.000	0.388***	0.118***	–	1.000	0.327***	0.100***
IADL	–	–	1.000	0.120***	–	–	1.000	0.100***	–	–	1.000	0.093***
Depression	–	–	–	1.000	–	–	–	1.000	–	–	–	1.000

–: data duplication.

BADL = basic activities of daily living, CHARLS = China Health and Retirement Longitudinal Study, CLD = chronic lung disease, ELSA = English Longitudinal Study of Ageing, HRS = Health and Retirement Study, IADL = instrumental activities of daily living.

*** *P* value < .001.

### 3.3. The mediating role of ADL dependence in the CLD–depression association

To elucidate the potential mediating role of BADL dependence in the association between CLD and the risk of depression, we first analyzed the relationship between CLD and depression within each dataset, adjusting for covariates (Model 1, Table 3). Model 1 displayed a significant association between CLD and depression (CHARLS: OR = 1.454, 95% CI: 1.212–1.743; ELSA: OR = 1.671, 95% CI: 1.164–2.399; HRS: OR = 1.619, 95% CI: 1.325–1.977) after covariate adjustment, a finding consistently validated across multiple datasets (Table 3, Supplementary Tables S3–S5, Supplemental Digital Content 3). Upon inclusion of BADL dependence as a mediator in Model 2, the association between CLD and depression (CHARLS: OR = 1.417, 95% CI: 1.180–1.700; ELSA: OR = 1.558, 95% CI: 1.081–2.245; HRS: OR = 1.530, 95% CI: 1.250–1.872) remained significant in all 3 datasets, and BADL dependence maintained a significant association with depression across all datasets (*P* < .05, Table 3). Similarly, when IADL dependence was added as a mediator in Model 3, the CLD‑depression association remained significant in all 3 datasets (CHARLS: OR = 1.425, 95% CI: 1.187–1.711; ELSA: OR = 1.594, 95% CI: 1.107–2.295; HRS: OR = 1.538, 95% CI: 1.257–1.883), and IADL dependence was also significantly associated with depression in all cohorts (CHARLS: OR = 1.760, 95% CI: 1.490–2.078; ELSA: OR = 2.248, 95% CI: 1.568–3.223; HRS: OR = 2.207, 95% CI: 1.785–2.729, Table 3).

**Table 3:** Associations of chronic lung disease and activities of daily living with depression.

Variable	Model 1		Model 2		Model 3	
	OR (95% CI)	*P* value	OR (95% CI)	*P* value	OR (95% CI)	*P* value
CHARLS						
CLD (reference = no)	1.454 (1.212–1.743)	<.001	1.417 (1.180–1.700)	<.001	1.425 (1.187–1.711)	<.001
BADL (reference = independence)	–		1.591 (1.326–1.910)	<.001	–	
IADL (reference = independence)	–		–		1.760 (1.490–2.078)	<.001
ELSA						
CLD (reference = no)	1.671 (1.164–2.399)	.005	1.558 (1.081–2.245)	.017	1.594 (1.107–2.295)	.012
BADL (reference = independence)	–		2.275 (1.764–2.934)	<.001	–	
IADL (reference = independence)	–		–		2.248 (1.568–3.223)	<.001
HRS						
CLD (reference = no)	1.619 (1.325–1.977)	<.001	1.530 (1.250–1.872)	<.001	1.538 (1.257–1.883)	<.001
BADL (reference = independence)	–		2.045 (1.698–2.464)	<.001	–	
IADL (reference = independence)	–		–		2.207 (1.785–2.729)	<.001

–: data duplication.

Model 1 was adjusted for covariates, including sex, age, alcohol consumption, smoking status, educational levels, marital status, hypertension, and diabetes.

Model 2 built upon Model 1 by incorporating BADL as a mediator.

Model 3 extended Model 1 by adding IADL as a mediator.

The results for covariates are presented in Supplementary Tables S3–S5, Supplemental Digital Content 3.

BADL = basic activities of daily living, CHARLS = China Health and Retirement Longitudinal Study, CI = confidence interval, CLD = chronic lung disease, ELSA = English Longitudinal Study of Ageing, HRS = Health and Retirement Study, IADL = instrumental activities of daily living, OR = odds ratio.

Subsequently, mediation analyses were conducted. The results suggested statistically significant mediation effects associated with both BADL dependence and IADL dependence across all datasets (Table 4). In the CHARLS cohort, the potential proportions of the total association between CLD and depression that were mediated by BADL dependence and IADL dependence were 7.8% (95% CI: 2.9%–17.3%) and 6.2% (95% CI: 0.9%–16.1%), respectively. In the ELSA cohort, these proportions were 13.7% (95% CI: 2.9%–55.4%) and 9.1% (95% CI: 1.9%–38.8%), respectively. In the HRS cohort, the potential mediated proportions for BADL dependence and IADL dependence were 10.1% (95% CI: 5.5%–22.7%) and 12.1% (95% CI: 4.8%–21.6%), respectively (Table 4).

**Table 4:** The results of mediation analysis.

Source	Total effect*	*P* value	Direct effect*	*P* value	Indirect effect*	*P* value	Proportion of mediation	*P* value
BADL								
CHARLS	0.077 (95% CI: 0.040–0.115)	<.001	0.006 (95% CI: 0.002–0.011)	<.001	0.071 (95% CI: 0.035–0.109)	<.001	0.078 (95% CI: 0.029–0.173)	<.001
ELSA	0.058 (95% CI: 0.010–0.110)	.012	0.008 (95% CI: 0.003–0.016)	.006	0.050 (95% CI: 0.003–0.101)	.040	0.137 (95% CI: 0.029–0.554)	.018
HRS	0.061 (95% CI: 0.033–0.093)	<.001	0.006 (95% CI: 0.004–0.011)	<.001	0.055 (95% CI: 0.027–0.086)	<.001	0.101 (95% CI: 0.055–0.227)	<.001
IADL								
CHARLS	0.077 (95% CI: 0.035–0.117)	<.001	0.005 (95% CI: 0.001–0.009)	.024	0.072 (95% CI: 0.031–0.111)	<.001	0.062 (95% CI: 0.009–0.161)	.024
ELSA	0.058 (95% CI: 0.013–0.107)	.010	0.005 (95% CI: 0.001–0.012)	.012	0.053 (95% CI: 0.008–0.100)	.028	0.091 (95% CI: 0.019–0.388)	.022
HRS	0.064 (95% CI: 0.033–0.092)	<.001	0.008 (95% CI: 0.003–0.011)	<.001	0.056 (95% CI: 0.027–0.085)	<.001	0.121 (95% CI: 0.048–0.216)	<.001

BADL = basic activities of daily living, CHARLS = China Health and Retirement Longitudinal Study, CI = confidence interval, ELSA = English Longitudinal Study of Ageing, HRS = Health and Retirement Study, IADL = instrumental activities of daily living.

* The effect in this table is the standardized effect.

### 3.4. Sensitivity analysis

Logistic regression models were applied to the imputed datasets, and the results demonstrated that the depressive effects of CLD and ADL dependence remained statistically significant (Supplementary Tables S6–S8, Supplemental Digital Content 4). Subsequently, mediation analyses were performed separately on each of the 20 imputed datasets. The mediation effects were significant across all datasets in 3 cohorts, indicating the robustness of the findings (Supplementary Table S9, Supplemental Digital Content 5).

## 4. Discussion

This study systematically investigated the association between CLD and depression among middle-aged and older adults using 3 large-scale population databases, including CHARLS, ELSA, and HRS. Particular attention was given to the potential mediating role of ADL dependence, including BADL dependence and IADL dependence. The results of this study indicate that, among middle-aged and elderly patients with CLD from different countries, BADL dependence and IADL dependence may be associated with the onset of depression through potential mediating pathways. It should be noted that, because CLD and dependence in ADL were measured concurrently at baseline, the temporal order between the exposure and mediator variables cannot be determined; therefore, this mediating relationship requires further validation.

In this study, the incident depression was 27.5% in CHARLS, 11.3% in ELSA, and 13.4% in HRS, which is lower compared with previous studies.^[27]^ It is worth noting that the rate of depression among the Chinese population is relatively high. In China, middle-aged and older adults are accustomed to assisting their younger children and actively participating in their lives, such as by caring for grandchildren and performing household chores.^[28,29]^ The participation of older adults in the US and the UK in this regard was lower than that of their counterparts in China. Additionally, the educational level in CHARLS is lower compared with that in the cohorts from the 2 other countries, hindering accurate recognition of depression and consequently exacerbating its symptoms.^[19]^

BADL and IADL are primary tools for physical function.^[13]^ When ADL dependence occurs, care is predominantly provided by their children.^[30]^ The combined effects of illness, feelings of guilt, and the accompanying functional disabilities may contribute to an increased incidence of depression within this population. Previous studies have identified ADL dependence as a significant risk factor for depression.^[15–18]^ Results from random forest models assessing depression risk among older adults in China and the US indicate that BADL dependence and IADL dependence are key mediators of urban–rural disparities in depression among Chinese elderly individuals, whereas such associations are not significant in American adults.^[31]^ A study focused on the Chinese population similarly demonstrated that BADL dependence and IADL dependence increase the risk of depression in middle-aged and older adults, with both prevalent and incident ADL dependence contributing to elevated depression risk.^[14]^ Additionally, 2 other studies within China have reported that functional disability precipitates depression.^[16,17]^ A recent multicenter study analyzed the dynamic relationship between ADL dependence and depression, demonstrating that improvements in ADL dependence are associated with a reduced risk of depression.^[14]^

Patients with CLD often exist in a state of inflammation; notably, even during stable phases, individuals with COPD exhibit a persistent low-grade inflammatory state.^[32]^ This inflammatory milieu has been shown to increase the risk of depression in COPD patients.^[32]^ Large-scale population analyses comparing CLD and non-CLD groups have demonstrated statistically significant differences in depression incidence between these groups.^[33]^ Tumor necrosis factor-alpha (TNF-α), a key pro-inflammatory cytokine, has been implicated in symptoms such as social withdrawal, weight loss, and increased behaviors associated with despair.^[34]^ Elevated expression of TNF-α also promotes catabolism and inhibits protein synthesis in skeletal muscle cells, thereby contributing to impaired physical function in the elderly population.^[35]^ Elevated levels of TNF-α have been observed in both patients with COPD and those with functional impairments.^[32,35]^ Therefore, pro-inflammatory cytokines may represent a common biological mechanism underlying the high prevalence of depression in patients with ADL dependence and CLD.

Our study indicates that ADL dependence serves as a potential mediator between CLD and depression. Previous research has shown that improving ADL can reduce the risk of depression.^[14]^ Notably, the mediating effect of dependence in both BADL and IADL in the pathway from CLD to depression was relatively modest across the 3 international cohorts, ranging only from 6.2% to 13.7%. Given the very small mediation proportions, ADL dependence explained only a minor fraction of the CLD–depression association. Thus, ADL-focused interventions are unlikely to yield meaningful clinical benefits for depression prevention in this population.

This study has several limitations. First, as public datasets were used, detailed clinical diagnostic information for the patients was unavailable, which may have affected the conclusions. Second, the covariates included in this study were limited, making it impossible to ascertain the influence of other unmeasured confounders on the results. Differences in socioeconomic and cultural factors, as well as variations in database measurement methods, may also lead to disparities. Additionally, factors such as frailty,^[36]^ physical activity,^[37]^ cognitive impairment,^[38]^ sleep disorders,^[39]^ social isolation,^[40]^ socioeconomic status,^[41]^ CLD severity, and medication use^[42]^ may also influence the conclusions of this study. Future research should further clarify the impacts of these differences. Third, although the mediating role of ADL dependence in depression among elderly patients with CLD was clarified in this study, whether improving ADL status can reduce the severity of depression remains uncertain. Fourth, different evaluation criteria were used when assessing the same indicator. For instance, different assessment methods were employed to evaluate participants’ depressive symptoms (8-item Center for Epidemiologic Studies Depression Scale vs 10-item Center for Epidemiologic Studies Depression Scale). For ADL dependence, we adopted the measure of voluntary control of urination as a substitute for free walking in the room in CHARLS. Utilizing a standardized assessment tool may help eliminate potential heterogeneity. We also did not analyze the impact of different subtypes of CLD and disease severity on potential mediating effects. Furthermore, in the present study, BADL and IADL were treated as binary dichotomous variables (dependence vs independence), which may reduce statistical power and discard clinically meaningful gradient information regarding the severity of functional impairment. Finally, self-reported health status was employed in this study, which may have introduced recall bias and thereby influenced the findings.

## 5. Conclusion

The findings of this study are consistent with a potential mediating role of ADL dependence in the association between CLD and incident depression.

## Acknowledgments

We gratefully acknowledge the research teams of CHARLS, ELSA, and HRS for providing access to their data. We would like to express our gratitude to Dr Qing-ao Xiao from Xiangya Hospital, Central South University, for his help with the statistical analysis and manuscript revisions.

## Author contributions

**Data curation:** Yu-Li Zhang, Wen-Bin Bai.

**Formal analysis:** Yu-Li Zhang.

**Investigation:** Yu-Li Zhang, Wen-Bin Bai, Jing-Xia Qin.

**Methodology:** Yu-Li Zhang, Jing-Xia Qin.

**Resources:** Yu-Li Zhang, Wen-Bin Bai.

**Project administration:** Wen-Bin Bai.

**Supervision:** Wen-Bin Bai.

**Funding acquisition:** Jing-Xia Qin.

**Software:** Yu-Li Zhang.

**Validation:** Yu-Li Zhang, Lei Qin, Wen-Bin Bai, Nian Shen, Jing-Xia Qin.

**Visualization:** Yu-Li Zhang, Wen-Bin Bai, Jing-Xia Qin.

**Writing – original draft:** Yu-Li Zhang, Wen-Bin Bai, Jing-Xia Qin.

**Writing – review & editing:** Lei Qin, Nian Shen, Jing-Xia Qin.


















