# Supplementary material for: ADL dependence may represent a potential pathway linking chronic lung disease and depression in the middle-aged and older adults: A prospective cross-national cohort study (STROBE)
Source: Medicine (Baltimore). 2026 Jul 3;105(27):e49589. doi: 10.1097/MD.0000000000049589 (PMC13337061; doi:10.1097/MD.0000000000049589)
Supplement: Supplementary file 1 [file medi-105-e49589-s001.docx]

**Table S1. The characteristics of participants in this study.**

| **Variables** | **CHARLS** | | | **ELSA** | | | **HRS** | | |
| --- | --- | --- | --- | --- | --- | --- | --- | --- | --- |
|  | **Non-depression**  **(n = 3927)** | **Depression**  **(n = 1491)** | ***P***  **value** | **Non-depression**  **(n = 4045)** | **Depression**  **(n = 515)** | ***P***  **value** | **Non-depression**  **(n = 7431)** | **Depression**  **(n = 1145)** | ***P***  **value** |
| Age, n (%) |  |  | 0.002 |  |  | 0.055 |  |  | 0.426 |
| ≤60 years | 1999 (50.9) | 689 (46.2) |  | 1009 (24.9) | 108 (21) |  | 2474 (33.3) | 367 (32.1) |  |
| >60 years | 1928 (49.1) | 802 (53.8) |  | 3036 (75.1) | 407 (79) |  | 4957 (66.7) | 778 (67.9) |  |
| Sex, n (%) |  |  | < 0.001 |  |  | < 0.001 |  |  | < 0.001 |
| Female | 1622 (41.3) | 835 (56) |  | 2059 (50.9) | 334 (64.9) |  | 4202 (56.5) | 756 (66) |  |
| Male | 2305 (58.7) | 656 (44) |  | 1986 (49.1) | 181 (35.1) |  | 3229 (43.5) | 389 (34) |  |
| Marital status, n (%) |  |  | 0.003 |  |  | < 0.001 |  |  | < 0.001 |
| Married | 3572 (91) | 1315 (88.2) |  | 1050 (26) | 201 (39) |  | 2621 (35.3) | 475 (41.5) |  |
| Other | 355 (9) | 176 (11.8) |  | 2995 (74) | 314 (61) |  | 4810 (64.7) | 670 (58.5) |  |
| Education status, n (%) |  |  | < 0.001 |  |  | < 0.001 |  |  | 0.004 |
| High school and below | 3818 (97.2) | 1482 (99.4) |  | 1900 (47) | 309 (60) |  | 3204 (43.1) | 546 (47.7) |  |
| College and above | 109 (2.8) | 9 (0.6) |  | 2145 (53) | 206 (40) |  | 4227 (56.9) | 599 (52.3) |  |
| Smoking status, n (%) |  |  | < 0.001 |  |  | 0.063 |  |  | 0.047 |
| No | 1903 (48.5) | 865 (58) |  | 1630 (40.3) | 185 (35.9) |  | 3528 (47.5) | 507 (44.3) |  |
| Yes | 2024 (51.5) | 626 (42) |  | 2415 (59.7) | 330 (64.1) |  | 3903 (52.5) | 638 (55.7) |  |
| Drinking status, n (%) |  |  | < 0.001 |  |  | < 0.001 |  |  | < 0.001 |
| No | 2321 (59.1) | 1012 (67.9) |  | 381 (9.4) | 93 (18.1) |  | 2845 (38.3) | 500 (43.7) |  |
| Yes | 1606 (40.9) | 479 (32.1) |  | 3664 (90.6) | 422 (81.9) |  | 4586 (61.7) | 645 (56.3) |  |
| Diabetes, n (%) |  |  | 0.397 |  |  | 0.216 |  |  | 0.117 |
| No | 3725 (94.9) | 1405 (94.2) |  | 3692 (91.3) | 461 (89.5) |  | 5854 (78.8) | 878 (76.7) |  |
| Yes | 202 (5.1) | 86 (5.8) |  | 353 (8.7) | 54 (10.5) |  | 1577 (21.2) | 267 (23.3) |  |
| Hypertension, n (%) |  |  | < 0.001 |  |  | < 0.001 |  |  | < 0.001 |
| No | 3032 (77.2) | 1075 (72.1) |  | 2498 (61.8) | 277 (53.8) |  | 3307 (44.5) | 437 (38.2) |  |
| Yes | 895 (22.8) | 416 (27.9) |  | 1547 (38.2) | 238 (46.2) |  | 4124 (55.5) | 708 (61.8) |  |
| CLD, n (%) |  |  | < 0.001 |  |  | < 0.001 |  |  | < 0.001 |
| No | 3513 (89.5) | 1279 (85.8) |  | 3872 (95.7) | 473 (91.8) |  | 6886 (92.7) | 1001 (87.4) |  |
| Yes | 414 (10.5) | 212 (14.2) |  | 173 (4.3) | 42 (8.2) |  | 545 (7.3) | 144 (12.6) |  |
| BADL, n (%) |  |  | < 0.001 |  |  | < 0.001 |  |  | < 0.001 |
| Independence | 3572 (91) | 1260 (84.5) |  | 3701 (91.5) | 414 (80.4) |  | 6874 (92.5) | 965 (84.3) |  |
| Dependence | 355 (9) | 231 (15.5) |  | 344 (8.5) | 101 (19.6) |  | 557 (7.5) | 180 (15.7) |  |
| IADL, n (%) |  |  | < 0.001 |  |  | < 0.001 |  |  | < 0.001 |
| Independence | 3497 (89.1) | 1191 (79.9) |  | 3915 (96.8) | 467 (90.7) |  | 7047 (94.8) | 1011 (88.3) |  |
| Dependence | 430 (10.9) | 300 (20.1) |  | 130 (3.2) | 48 (9.3) |  | 384 (5.2) | 134 (11.7) |  |

*Abbreviations*: BADL = Basic activities of daily living; CLD = Chronic lung diseases; IADL = instrumental activities of daily living; CHARLS = China Health and Retirement Longitudinal Study; ELSA = English Longitudinal Study of Ageing; HRS = Health and Retirement Study.
