# Supplementary material for: ADL dependence may represent a potential pathway linking chronic lung disease and depression in the middle-aged and older adults: A prospective cross-national cohort study (STROBE)
Source: Medicine (Baltimore). 2026 Jul 3;105(27):e49589. doi: 10.1097/MD.0000000000049589 (PMC13337061; doi:10.1097/MD.0000000000049589)
Supplement: Supplementary file 2 [file medi-105-e49589-s002.docx]

**Table S2. The characteristics of participants between included and excluded in this study.**

| **Variables** | **CHARLS** | | | **ELSA** | | | **HRS** | | |
| --- | --- | --- | --- | --- | --- | --- | --- | --- | --- |
|  | **Excluded**  **(n = 15677)** | **Included**  **(n = 5418)** | ***P***  **value** | **Excluded**  **(n = 5106)** | **Included**  **(n = 4560)** | ***P***  **value** | **Excluded**  **(n = 10171)** | **Included**  **(n = 8576)** | ***P***  **value** |
| Depression, n (%) |  |  | < 0.001 |  |  | < 0.001 |  |  | < 0.001 |
| Non-depression | 7837 (55.2) | 3927 (72.5) |  | 2681 (60.3) | 4045 (88.7) |  | 5141 (56.4) | 7431 (86.6) |  |
| Depression | 6356 (44.8) | 1491 (27.5) |  | 1762 (39.7) | 515 (11.3) |  | 3979 (43.6) | 1145 (13.4) |  |
| Age, n (%) |  |  | < 0.001 |  |  | < 0.001 |  |  | < 0.001 |
| ≤60 years | 9265 (59.5) | 2688 (49.6) |  | 1516 (29.7) | 1117 (24.5) |  | 2975 (29.2) | 2841 (33.1) |  |
| >60 years | 6315 (40.5) | 2730 (50.4) |  | 3590 (70.3) | 3443 (75.5) |  | 7196 (70.8) | 5735 (66.9) |  |
| Sex, n (%) |  |  | < 0.001 |  |  | < 0.001 |  |  | 0.004 |
| Female | 8572 (54.7) | 2457 (45.3) |  | 2975 (58.3) | 2393 (52.5) |  | 6090 (59.9) | 4958 (57.8) |  |
| Male | 7100 (45.3) | 2961 (54.7) |  | 2131 (41.7) | 2167 (47.5) |  | 4081 (40.1) | 3618 (42.2) |  |
| Marital status, n (%) |  |  | < 0.001 |  |  | < 0.001 |  |  | < 0.001 |
| Married | 13405 (85.8) | 4887 (90.2) |  | 1983 (38.9) | 1251 (27.4) |  | 5175 (51) | 3096 (36.1) |  |
| Other | 2211 (14.2) | 531 (9.8) |  | 3121 (61.1) | 3309 (72.6) |  | 4977 (49) | 5480 (63.9) |  |
| Education status, n (%) |  |  | 0.244 |  |  | < 0.001 |  |  | < 0.001 |
| High school and below | 15358 (98.1) | 5300 (97.8) |  | 2632 (62.9) | 2209 (48.4) |  | 6010 (59.1) | 3750 (43.7) |  |
| College and above | 299 (1.9) | 118 (2.2) |  | 1550 (37.1) | 2351 (51.6) |  | 4156 (40.9) | 4826 (56.3) |  |
| Smoking status, n (%) |  |  | < 0.001 |  |  | < 0.001 |  |  | < 0.001 |
| No | 9127 (58.8) | 2768 (51.1) |  | 1771 (36) | 1815 (39.8) |  | 4216 (41.9) | 4035 (47) |  |
| Yes | 6386 (41.2) | 2650 (48.9) |  | 3154 (64) | 2745 (60.2) |  | 5850 (58.1) | 4541 (53) |  |
| Drinking status, n (%) |  |  | < 0.001 |  |  | < 0.001 |  |  | < 0.001 |
| No | 10151 (65.8) | 3333 (61.5) |  | 585 (16.9) | 474 (10.4) |  | 5362 (52.7) | 3345 (39) |  |
| Yes | 5277 (34.2) | 2085 (38.5) |  | 2886 (83.1) | 4086 (89.6) |  | 4805 (47.3) | 5231 (61) |  |
| Diabetes, n (%) |  |  | 0.018 |  |  | < 0.001 |  |  | < 0.001 |
| No | 10076 (93.7) | 5130 (94.7) |  | 4365 (85.5) | 4153 (91.1) |  | 7311 (71.9) | 6732 (78.5) |  |
| Yes | 673 (6.3) | 288 (5.3) |  | 741 (14.5) | 407 (8.9) |  | 2860 (28.1) | 1844 (21.5) |  |
| Hypertension, n (%) |  |  | < 0.001 |  |  | < 0.001 |  |  | < 0.001 |
| No | 7141 (72.2) | 4107 (75.8) |  | 2774 (54.3) | 2775 (60.9) |  | 3444 (33.9) | 3744 (43.7) |  |
| Yes | 2749 (27.8) | 1311 (24.2) |  | 2332 (45.7) | 1785 (39.1) |  | 6727 (66.1) | 4832 (56.3) |  |
| CLD, n (%) |  |  | < 0.001 |  |  | < 0.001 |  |  | < 0.001 |
| No | 9804 (84.4) | 4792 (88.4) |  | 4696 (92) | 4345 (95.3) |  | 8479 (83.4) | 7887 (92) |  |
| Yes | 1809 (15.6) | 626 (11.6) |  | 410 (8) | 215 (4.7) |  | 1692 (16.6) | 689 (8) |  |
| BADL, n (%) |  |  | < 0.001 |  |  | < 0.001 |  |  | < 0.001 |
| Independence | 11396 (77.1) | 4832 (89.2) |  | 3879 (76) | 4115 (90.2) |  | 7056 (69.5) | 7839 (91.4) |  |
| Dependence | 3380 (22.9) | 586 (10.8) |  | 1225 (24) | 445 (9.8) |  | 3098 (30.5) | 737 (8.6) |  |
| IADL, n (%) |  |  | < 0.001 |  |  | < 0.001 |  |  | < 0.001 |
| Independence | 11416 (73.6) | 4688 (86.5) |  | 4162 (81.5) | 4382 (96.1) |  | 7211 (71) | 8058 (94) |  |
| Dependence | 4085 (26.4) | 730 (13.5) |  | 942 (18.5) | 178 (3.9) |  | 2943 (29) | 518 (6) |  |

*Abbreviations*: BADL = Basic activities of daily living; CLD = Chronic lung diseases; IADL = instrumental activities of daily living; CHARLS = China Health and Retirement Longitudinal Study; ELSA = English Longitudinal Study of Ageing; HRS = Health and Retirement Study.
