# Supplementary material for: ADL dependence may represent a potential pathway linking chronic lung disease and depression in the middle-aged and older adults: A prospective cross-national cohort study (STROBE)
Source: Medicine (Baltimore). 2026 Jul 3;105(27):e49589. doi: 10.1097/MD.0000000000049589 (PMC13337061; doi:10.1097/MD.0000000000049589)
Supplement: Supplementary file 3 [file medi-105-e49589-s003.docx]

**Table S3. Associations of chronic lung disease and activities of daily living with depression in China Health and Retirement Longitudinal Study.**

| **Variable** | **Model 1** | | **Model 2** | | **Model 3** | |
| --- | --- | --- | --- | --- | --- | --- |
|  | **OR (95%CI)** | ***P* value** | **OR (95%CI)** | ***P* value** | **OR (95%CI)** | ***P* value** |
| CLD |  |  |  |  |  |  |
| No | Ref |  | Ref |  | Ref |  |
| Yes | 1.454 (1.212-1.743) | <0.001 | 1.417 (1.180-1.700) | <0.001 | 1.425 (1.187-1.711) | <0.001 |
| BADL |  |  |  |  |  |  |
| Independence | -- |  | Ref |  | -- |  |
| Dependence | -- |  | 1.591 (1.326-1.910) | <0.001 | -- |  |
| IADL |  |  |  |  |  |  |
| Independence | -- |  | -- |  | Ref |  |
| Dependence | -- |  | -- |  | 1.760 (1.490-2.078) | <0.001 |
| Age |  |  |  |  |  |  |
| ≤60 years | Ref |  | Ref |  | Ref |  |
| >60 years | 1.170 (1.033-1.326) | 0.013 | 1.133 (0.999-1.285) | 0.052 | 1.115 (0.983-1.265) | 0.090 |
| Sex |  |  |  |  |  |  |
| Female | Ref |  | Ref |  | Ref |  |
| Male | 0.535 (0.441-0.649) | <0.001 | 0.546 (0.450-0.663) | <0.001 | 0.559 (0.460-0.679) | <0.001 |
| Education status |  |  |  |  |  |  |
| High school and below | Ref |  | Ref |  | Ref |  |
| College and above | 0.250 (0.126-0.496) | <0.001 | 0.261 (0.131-0.518) | <0.001 | 0.264 (0.133-0.525) | <0.001 |
| Marital status |  |  |  |  |  |  |
| Married | Ref |  | Ref |  | Ref |  |
| Other | 1.138 (0.933-1.387) | 0.202 | 1.120 (0.918-1.366) | 0.264 | 1.103 (0.903-1.347) | 0.336 |
| Diabetes |  |  |  |  |  |  |
| No | Ref |  | Ref |  | Ref |  |
| Yes | 1.031 (0.789-1.349) | 0.821 | 1.020 (0.779-1.335) | 0.884 | 1.028 (0.785-1.347) | 0.839 |
| Hypertension |  |  |  |  |  |  |
| No | Ref |  | Ref |  | Ref |  |
| Yes | 1.238 (1.076-1.425) | 0.003 | 1.211 (1.051-1.395) | 0.008 | 1.211 (1.051-1.395) | 0.008 |
| Drinking status |  |  |  |  |  |  |
| No | Ref |  | Ref |  | Ref |  |
| Yes | 0.923 (0.799-1.065) | 0.273 | 0.923 (0.799-1.066) | 0.275 | 0.930 (0.805-1.074) | 0.323 |
| Smoking status |  |  |  |  |  |  |
| No | Ref |  | Ref |  | Ref |  |
| Yes | 1.100 (0.913-1.325) | 0.317 | 1.098 (0.911-1.322) | 0.328 | 1.091 (0.905-1.315) | 0.363 |

*Abbreviations*: BADL = Basic activities of daily living; IADL = Instrumental activities of daily living; OR = Odds ratio; CI = Confidence interval; CLD = Chronic lung disease.

Model 1 was adjusted for covariates including sex, age, alcohol consumption, smoking status, educational attainment, marital status, hypertension, and diabetes.

Model 2 built upon Model 1 by incorporating BADL as a mediator.

Model 3 extended Model 1 by adding IADL as a mediator.
