# Supplementary material for: ADL dependence may represent a potential pathway linking chronic lung disease and depression in the middle-aged and older adults: A prospective cross-national cohort study (STROBE)
Source: Medicine (Baltimore). 2026 Jul 3;105(27):e49589. doi: 10.1097/MD.0000000000049589 (PMC13337061; doi:10.1097/MD.0000000000049589)
Supplement: Supplementary file 4 [file medi-105-e49589-s004.docx]

**Table S6. Associations of chronic lung disease and activities of daily living with depression in China Health and Retirement Longitudinal Study (Imputed data).**

| **Variable** | **Model 1** | | **Model 2** | | **Model 3** | |
| --- | --- | --- | --- | --- | --- | --- |
|  | **OR (95%CI)** | ***P* value** | **OR (95%CI)** | ***P* value** | **OR (95%CI)** | ***P* value** |
| CLD |  |  |  |  |  |  |
| No | Ref |  | Ref |  | Ref |  |
| Yes | 1.331 (1.119-1.583) | 0.001 | 1.296 (1.090-1.541) | 0.004 | 1.307 (1.098-1.555) | 0.003 |
| BADL |  |  |  |  |  |  |
| Independence | -- |  | Ref |  | -- |  |
| Dependence | -- |  | 1.616 (1.383-1.888) | <0.001 | -- |  |
| IADL |  |  |  |  |  |  |
| Independence | -- |  | -- |  | Ref |  |
| Dependence | -- |  | -- |  | 1.712 (1.484-1.976) | <0.001 |
| Age |  |  |  |  |  |  |
| ≤60 years | Ref |  | Ref |  | Ref |  |
| >60 years | 1.206 (1.082-1.344) | 0.001 | 1.167 (1.046-1.301) | 0.006 | 1.136 (1.018-1.268) | 0.023 |
| Sex |  |  |  |  |  |  |
| Female | Ref |  | Ref |  | Ref |  |
| Male | 0.532 (0.451-0.628) | <0.001 | 0.544 (0.461-0.642) | <0.001 | 0.558 (0.472-0.659) | <0.001 |
| Education status |  |  |  |  |  |  |
| High school and below | Ref |  | Ref |  | Ref |  |
| College and above | 0.287 (0.159-0.518) | <0.001 | 0.301 (0.167-0.544) | <0.001 | 0.304 (0.169-0.548) | <0.001 |
| Marital status |  |  |  |  |  |  |
| Married | Ref |  | Ref |  | Ref |  |
| Other | 1.080 (0.899-1.297) | 0.413 | 1.053 (0.875-1.268) | 0.584 | 1.037 (0.861-1.250) | 0.702 |
| Diabetes |  |  |  |  |  |  |
| No | Ref |  | Ref |  | Ref |  |
| Yes | 1.005 (0.784-1.289) | 0.966 | 0.991 (0.773-1.271) | 0.946 | 0.993 (0.774-1.273) | 0.954 |
| Hypertension |  |  |  |  |  |  |
| No | Ref |  | Ref |  | Ref |  |
| Yes | 1.192 (1.053-1.349) | 0.006 | 1.162 (1.026-1.316) | 0.019 | 1.168 (1.032-1.323) | 0.014 |
| Drinking status |  |  |  |  |  |  |
| No | Ref |  | Ref |  | Ref |  |
| Yes | 0.884 (0.778-1.005) | 0.061 | 0.885 (0.778-1.006) | 0.063 | 0.901 (0.792-1.026) | 0.116 |
| Smoking status |  |  |  |  |  |  |
| No | Ref |  | Ref |  | Ref |  |
| Yes | 1.132 (0.962-1.332) | 0.136 | 1.129 (0.959-1.329) | 0.145 | 1.118 (0.949-1.316) | 0.182 |

*Abbreviations*: BADL = Basic activities of daily living; IADL = Instrumental activities of daily living; OR = Odds ratio; CI = Confidence interval; CLD = Chronic lung disease.

Model 1 was adjusted for covariates including sex, age, alcohol consumption, smoking status, educational attainment, marital status, hypertension, and diabetes.

Model 2 built upon Model 1 by incorporating BADL as a mediator.

Model 3 extended Model 1 by adding IADL as a mediator.
