# Supplementary material for: ADL dependence may represent a potential pathway linking chronic lung disease and depression in the middle-aged and older adults: A prospective cross-national cohort study (STROBE)
Source: Medicine (Baltimore). 2026 Jul 3;105(27):e49589. doi: 10.1097/MD.0000000000049589 (PMC13337061; doi:10.1097/MD.0000000000049589)
Supplement: Supplementary file 5 [file medi-105-e49589-s005.docx]

**Table S9. The results of mediation analysis (Imputed data).**

| Source | Total Effect^#^ | *P* value | Direct effect^#^ | *P* value | Indirect effect^#^ | *P* value | Proportion of mediation | *P* value |
| --- | --- | --- | --- | --- | --- | --- | --- | --- |
| **BADL** |  |  |  |  |  |  |  |  |
| CHARLS | 0.058  (95%CI: 0.031 - 0.086) | <0.001 | 0.006  (95%CI: 0.003 - 0.008) | <0.001 | 0.052  (95%CI: 0.026 - 0.079) | <0.001 | 0.101  (95%CI: 0.051 - 0.152) | <0.001 |
| ELSA | 0.049  (95%CI: 0.023 - 0.075) | <0.001 | 0.008  (95%CI: 0.006 - 0.011) | <0.001 | 0.041  (95%CI: 0.015 - 0.066) | 0.002 | 0.175  (95%CI: 0.075 - 0.276) | 0.001 |
| HRS | 0.051  (95%CI: 0.032 - 0.069) | <0.001 | 0.008  (95%CI: 0.006 - 0.01) | <0.001 | 0.043  (95%CI: 0.024 - 0.062) | <0.001 | 0.155  (95%CI: 0.086 - 0.224) | <0.001 |
| **IADL** |  |  |  |  |  |  |  |  |
| CHARLS | 0.058  (95%CI: 0.03 - 0.086) | <0.001 | 0.004  (95%CI: 0.002 - 0.007) | 0.002 | 0.054  (95%CI: 0.027 - 0.081) | <0.001 | 0.074  (95%CI: 0.029 - 0.119) | 0.001 |
| ELSA | 0.049  (95%CI: 0.023 - 0.074) | <0.001 | 0.008  (95%CI: 0.004 - 0.011) | <0.001 | 0.041  (95%CI: 0.016 - 0.066) | 0.001 | 0.159  (95%CI: 0.068 - 0.251) | 0.001 |
| HRS | 0.051  (95%CI: 0.032 - 0.069) | <0.001 | 0.006  (95%CI: 0.004 - 0.009) | <0.001 | 0.044  (95%CI: 0.026 - 0.062) | <0.001 | 0.129  (95%CI: 0.081 - 0.176) | <0.001 |

**^#^**The effect in this table is the standardized effect.

*Abbreviations*: BADL = Basic activities of daily living; IADL = instrumental activities of daily living.
