# Supplementary material for: ADL dependence may represent a potential pathway linking chronic lung disease and depression in the middle-aged and older adults: A prospective cross-national cohort study (STROBE)
Source: Medicine (Baltimore). 2026 Jul 3;105(27):e49589. doi: 10.1097/MD.0000000000049589 (PMC13337061; doi:10.1097/MD.0000000000049589)
Supplement: Supplementary file 6 [file medi-105-e49589-s006.docx]

**Table S4. Associations of chronic lung disease and activities of daily living with depression in English Longitudinal Study of Ageing.**

| **Variable** | **Model 1** | | **Model 2** | | **Model 3** | |
| --- | --- | --- | --- | --- | --- | --- |
|  | **OR (95%CI)** | ***P* value** | **OR (95%CI)** | ***P* value** | **OR (95%CI)** | ***P* value** |
| CLD |  |  |  |  |  |  |
| No | Ref |  | Ref |  | Ref |  |
| Yes | 1.671 (1.164-2.399) | 0.005 | 1.558 (1.081-2.245) | 0.017 | 1.594 (1.107-2.295) | 0.012 |
| BADL |  |  |  |  |  |  |
| Independence | -- |  | Ref |  | -- |  |
| Dependence | -- |  | 2.275 (1.764-2.934) | <0.001 | -- |  |
| IADL |  |  |  |  |  |  |
| Independence | -- |  | -- |  | Ref |  |
| Dependence | -- |  | -- |  | 2.248 (1.568-3.223) | <0.001 |
| Age |  |  |  |  |  |  |
| ≤60 years | Ref |  | Ref |  | Ref |  |
| >60 years | 1.107 (0.879-1.395) | 0.388 | 1.081 (0.857-1.363) | 0.512 | 1.097 (0.870-1.383) | 0.433 |
| Sex |  |  |  |  |  |  |
| Female | Ref |  | Ref |  | Ref |  |
| Male | 0.616 (0.504-0.752) | <0.001 | 0.606 (0.496-0.741) | <0.001 | 0.626 (0.512-0.765) | <0.001 |
| Education status |  |  |  |  |  |  |
| High school and below | Ref |  | Ref |  | Ref |  |
| College and above | 0.710 (0.585-0.861) | 0.001 | 0.720 (0.593-0.874) | 0.001 | 0.722 (0.594-0.876) | 0.001 |
| Marital status |  |  |  |  |  |  |
| Married | Ref |  | Ref |  | Ref |  |
| Other | 0.656 (0.539-0.799) | <0.001 | 0.660 (0.541-0.804) | <0.001 | 0.661 (0.542-0.805) | <0.001 |
| Diabetes |  |  |  |  |  |  |
| No | Ref |  | Ref |  | Ref |  |
| Yes | 1.055 (0.769-1.447) | 0.740 | 0.985 (0.716-1.356) | 0.927 | 1.030 (0.750-1.415) | 0.855 |
| Hypertension |  |  |  |  |  |  |
| No | Ref |  | Ref |  | Ref |  |
| Yes | 1.297 (1.070-1.573) | 0.008 | 1.249 (1.028-1.517) | 0.025 | 1.265 (1.042-1.536) | 0.017 |
| Drinking status |  |  |  |  |  |  |
| No | Ref |  | Ref |  | Ref |  |
| Yes | 0.584 (0.452-0.755) | <0.001 | 0.630 (0.485-0.817) | 0.001 | 0.622 (0.479-0.807) | <0.001 |
| Smoking status |  |  |  |  |  |  |
| No | Ref |  | Ref |  | Ref |  |
| Yes | 1.185 (0.973-1.443) | 0.092 | 1.147 (0.940-1.399) | 0.176 | 1.172 (0.962-1.428) | 0.116 |

*Abbreviations*: BADL = Basic activities of daily living; IADL = Instrumental activities of daily living; OR = Odds ratio; CI = Confidence interval; CLD = Chronic lung disease.

Model 1 was adjusted for covariates including sex, age, alcohol consumption, smoking status, educational attainment, marital status, hypertension, and diabetes.

Model 2 built upon Model 1 by incorporating BADL as a mediator.

Model 3 extended Model 1 by adding IADL as a mediator.
