# Supplementary material for: ADL dependence may represent a potential pathway linking chronic lung disease and depression in the middle-aged and older adults: A prospective cross-national cohort study (STROBE)
Source: Medicine (Baltimore). 2026 Jul 3;105(27):e49589. doi: 10.1097/MD.0000000000049589 (PMC13337061; doi:10.1097/MD.0000000000049589)
Supplement: Supplementary file 7 [file medi-105-e49589-s007.docx]

**Table S5. Associations of chronic lung disease and activities of daily living with depression in Health and Retirement Study.**

| **Variable** | **Model 1** | | **Model 2** | | **Model 3** | |
| --- | --- | --- | --- | --- | --- | --- |
|  | **OR (95%CI)** | ***P* value** | **OR (95%CI)** | ***P* value** | **OR (95%CI)** | ***P* value** |
| CLD |  |  |  |  |  |  |
| No | Ref |  | Ref |  | Ref |  |
| Yes | 1.619 (1.325-1.977) | <0.001 | 1.530 (1.250-1.872) | <0.001 | 1.538 (1.257-1.883) | <0.001 |
| BADL |  |  |  |  |  |  |
| Independence | -- |  | Ref |  | -- |  |
| Dependence | -- |  | 2.045 (1.698-2.464) | <0.001 | -- |  |
| IADL |  |  |  |  |  |  |
| Independence | -- |  | -- |  | Ref |  |
| Dependence | -- |  | -- |  | 2.207 (1.785-2.729) | <0.001 |
| Age |  |  |  |  |  |  |
| ≤60 years | Ref |  | Ref |  | Ref |  |
| >60 years | 0.966 (0.842-1.108) | 0.621 | 0.953 (0.831-1.094) | 0.496 | 0.983 (0.857-1.128) | 0.805 |
| Sex |  |  |  |  |  |  |
| Female | Ref |  | Ref |  | Ref |  |
| Male | 0.691 (0.603-0.792) | <0.001 | 0.693 (0.604-0.795) | <0.001 | 0.680 (0.593-0.780) | <0.001 |
| Education status |  |  |  |  |  |  |
| High school and below | Ref |  | Ref |  | Ref |  |
| College and above | 0.912 (0.802-1.037) | 0.160 | 0.937 (0.823-1.066) | 0.324 | 0.936 (0.822-1.065) | 0.313 |
| Marital status |  |  |  |  |  |  |
| Married | Ref |  | Ref |  | Ref |  |
| Other | 0.869 (0.762-0.991) | 0.036 | 0.894 (0.783-1.019) | 0.094 | 0.888 (0.778-1.012) | 0.076 |
| Diabetes |  |  |  |  |  |  |
| No | Ref |  | Ref |  | Ref |  |
| Yes | 1.045 (0.896-1.218) | 0.579 | 1.001 (0.857-1.170) | 0.985 | 1.023 (0.876-1.194) | 0.778 |
| Hypertension |  |  |  |  |  |  |
| No | Ref |  | Ref |  | Ref |  |
| Yes | 1.240 (1.084-1.418) | 0.002 | 1.213 (1.060-1.388) | 0.005 | 1.218 (1.064-1.394) | 0.004 |
| Drinking status |  |  |  |  |  |  |
| No | Ref |  | Ref |  | Ref |  |
| Yes | 0.875 (0.766-0.999) | 0.048 | 0.900 (0.787-1.028) | 0.121 | 0.901 (0.788-1.030) | 0.126 |
| Smoking status |  |  |  |  |  |  |
| No | Ref |  | Ref |  | Ref |  |
| Yes | 1.169 (1.026-1.333) | 0.019 | 1.161 (1.018-1.324) | 0.026 | 1.158 (1.016-1.321) | 0.028 |

*Abbreviations*: BADL = Basic activities of daily living; IADL = Instrumental activities of daily living; OR = Odds ratio; CI = Confidence interval; CLD = Chronic lung disease.

Model 1 was adjusted for covariates including sex, age, alcohol consumption, smoking status, educational attainment, marital status, hypertension, and diabetes.

Model 2 built upon Model 1 by incorporating BADL as a mediator.

Model 3 extended Model 1 by adding IADL as a mediator.
