# Supplementary material for: ADL dependence may represent a potential pathway linking chronic lung disease and depression in the middle-aged and older adults: A prospective cross-national cohort study (STROBE)
Source: Medicine (Baltimore). 2026 Jul 3;105(27):e49589. doi: 10.1097/MD.0000000000049589 (PMC13337061; doi:10.1097/MD.0000000000049589)
Supplement: Supplementary file 8 [file medi-105-e49589-s008.docx]

**Table S7. Associations of chronic lung disease and activities of daily living with depression in English Longitudinal Study of Ageing (Imputed data).**

| **Variable** | **Model 1** | | **Model 2** | | **Model 3** | |
| --- | --- | --- | --- | --- | --- | --- |
|  | **OR (95%CI)** | ***P* value** | **OR (95%CI)** | ***P* value** | **OR (95%CI)** | ***P* value** |
| CLD |  |  |  |  |  |  |
| No | Ref |  | Ref |  | Ref |  |
| Yes | 1.518 (1.110-2.076) | 0.009 | 1.419 (1.034-1.950) | 0.031 | 1.426 (1.040-1.955) | 0.028 |
| BADL |  |  |  |  |  |  |
| Independence | -- |  | Ref |  | -- |  |
| Dependence | -- |  | 2.050 (1.652-2.544) | <0.001 | -- |  |
| IADL |  |  |  |  |  |  |
| Independence | -- |  | -- |  | Ref |  |
| Dependence | -- |  | -- |  | 1.962 (1.476-2.607) | <0.001 |
| Age |  |  |  |  |  |  |
| ≤60 years | Ref |  | Ref |  | Ref |  |
| >60 years | 1.058 (0.873-1.283) | 0.564 | 1.020 (0.840-1.238) | 0.840 | 1.035 (0.853-1.256) | 0.728 |
| Sex |  |  |  |  |  |  |
| Female | Ref |  | Ref |  | Ref |  |
| Male | 0.599 (0.507-0.708) | <0.001 | 0.592 (0.500-0.700) | <0.001 | 0.608 (0.514-0.718) | <0.001 |
| Education status |  |  |  |  |  |  |
| High school and below | Ref |  | Ref |  | Ref |  |
| College and above | 0.744 (0.619-0.895) | 0.002 | 0.763 (0.634-0.918) | 0.005 | 0.758 (0.631-0.911) | 0.004 |
| Marital status |  |  |  |  |  |  |
| Married | Ref |  | Ref |  | Ref |  |
| Other | 0.672 (0.566-0.798) | <0.001 | 0.685 (0.576-0.814) | <0.001 | 0.689 (0.580-0.819) | <0.001 |
| Diabetes |  |  |  |  |  |  |
| No | Ref |  | Ref |  | Ref |  |
| Yes | 1.130 (0.869-1.471) | 0.362 | 1.044 (0.801-1.360) | 0.751 | 1.102 (0.845-1.437) | 0.473 |
| Hypertension |  |  |  |  |  |  |
| No | Ref |  | Ref |  | Ref |  |
| Yes | 1.240 (1.042-1.474) | 0.016 | 1.182 (0.992-1.408) | 0.062 | 1.209 (1.016-1.439) | 0.033 |
| Drinking status |  |  |  |  |  |  |
| No | Ref |  | Ref |  | Ref |  |
| Yes | 0.606 (0.480-0.764) | <0.001 | 0.659 (0.520-0.837) | 0.001 | 0.655 (0.517-0.830) | 0.001 |
| Smoking status |  |  |  |  |  |  |
| No | Ref |  | Ref |  | Ref |  |
| Yes | 1.116 (0.941-1.324) | 0.207 | 1.101 (0.928-1.307) | 0.270 | 1.112 (0.937-1.319) | 0.225 |

*Abbreviations*: BADL = Basic activities of daily living; IADL = Instrumental activities of daily living; OR = Odds ratio; CI = Confidence interval; CLD = Chronic lung disease.

Model 1 was adjusted for covariates including sex, age, alcohol consumption, smoking status, educational attainment, marital status, hypertension, and diabetes.

Model 2 built upon Model 1 by incorporating BADL as a mediator.

Model 3 extended Model 1 by adding IADL as a mediator.
