# Supplementary material for: ADL dependence may represent a potential pathway linking chronic lung disease and depression in the middle-aged and older adults: A prospective cross-national cohort study (STROBE)
Source: Medicine (Baltimore). 2026 Jul 3;105(27):e49589. doi: 10.1097/MD.0000000000049589 (PMC13337061; doi:10.1097/MD.0000000000049589)
Supplement: Supplementary file 9 [file medi-105-e49589-s009.docx]

**Table S8. Associations of chronic lung disease and activities of daily living with depression in Health and Retirement Study (Imputed data).**

| **Variable** | **Model 1** | | **Model 2** | | **Model 3** | |
| --- | --- | --- | --- | --- | --- | --- |
|  | **OR (95%CI)** | ***P* value** | **OR (95%CI)** | ***P* value** | **OR (95%CI)** | ***P* value** |
| CLD |  |  |  |  |  |  |
| No | Ref |  | Ref |  | Ref |  |
| Yes | 1.485 (1.245-1.770) | <0.001 | 1.395 (1.167-1.667) | <0.001 | 1.409 (1.180-1.682) | <0.001 |
| BADL |  |  |  |  |  |  |
| Independence | -- |  | Ref |  | -- |  |
| Dependence | -- |  | 1.860 (1.555-2.225) | <0.001 | -- |  |
| IADL |  |  |  |  |  |  |
| Independence | -- |  | -- |  | Ref |  |
| Dependence | -- |  | -- |  | 1.864 (1.555-2.234) | <0.001 |
| Age |  |  |  |  |  |  |
| ≤60 years | Ref |  | Ref |  | Ref |  |
| >60 years | 1.007 (0.879-1.154) | 0.921 | 0.976 (0.850-1.119) | 0.726 | 0.989 (0.862-1.134) | 0.869 |
| Sex |  |  |  |  |  |  |
| Female | Ref |  | Ref |  | Ref |  |
| Male | 0.706 (0.620-0.805) | <0.001 | 0.708 (0.621-0.807) | <0.001 | 0.700 (0.614-0.798) | <0.001 |
| Education status |  |  |  |  |  |  |
| High school and below | Ref |  | Ref |  | Ref |  |
| College and above | 0.910 (0.798-1.039) | 0.166 | 0.930 (0.814-1.061) | 0.280 | 0.932 (0.816-1.064) | 0.300 |
| Marital status |  |  |  |  |  |  |
| Married | Ref |  | Ref |  | Ref |  |
| Other | 0.880 (0.775-0.999) | 0.050 | 0.918 (0.808-1.043) | 0.189 | 0.913 (0.805-1.036) | 0.158 |
| Diabetes |  |  |  |  |  |  |
| No | Ref |  | Ref |  | Ref |  |
| Yes | 1.055 (0.904-1.232) | 0.498 | 1.019 (0.872-1.191) | 0.809 | 1.032 (0.884-1.205) | 0.688 |
| Hypertension |  |  |  |  |  |  |
| No | Ref |  | Ref |  | Ref |  |
| Yes | 1.203 (1.053-1.375) | 0.007 | 1.173 (1.024-1.342) | 0.022 | 1.179 (1.030-1.349) | 0.018 |
| Drinking status |  |  |  |  |  |  |
| No | Ref |  | Ref |  | Ref |  |
| Yes | 0.866 (0.769-0.975) | 0.018 | 0.895 (0.794-1.008) | 0.068 | 0.905 (0.804-1.019) | 0.101 |
| Smoking status |  |  |  |  |  |  |
| No | Ref |  | Ref |  | Ref |  |
| Yes | 1.120 (0.985-1.274) | 0.086 | 1.114 (0.980-1.268) | 0.102 | 1.114 (0.979-1.268) | 0.104 |

*Abbreviations*: BADL = Basic activities of daily living; IADL = Instrumental activities of daily living; OR = Odds ratio; CI = Confidence interval; CLD = Chronic lung disease.

Model 1 was adjusted for covariates including sex, age, alcohol consumption, smoking status, educational attainment, marital status, hypertension, and diabetes.

Model 2 built upon Model 1 by incorporating BADL as a mediator.

Model 3 extended Model 1 by adding IADL as a mediator.
